# Electrical Remodeling of Ventricular Repolarization Abnormality after Treatment in Pheochromocytoma: U Wave Finding in a Retrospective Analysis

**DOI:** 10.1155/2019/2605323

**Published:** 2019-04-10

**Authors:** Giuseppe Di Stolfo, Sandra Mastroianno, Angela Maggio, Giovanni De Luca, Domenico R. Potenza, Mauro Pellegrino Salvatori, Aldo Russo

**Affiliations:** ^1^Cardiology Unit, Cardiovascular Department, Fondazione IRCCS Casa Sollievo della Sofferenza, San Giovanni Rotondo (FG), Italy; ^2^Pediatric Oncohematology Unit, Maternal and Child Developmental Age Department, Fondazione IRCCS Casa Sollievo della Sofferenza, San Giovanni Rotondo (FG), Italy

## Abstract

**Background:**

Pheochromocytoma is a rare neuroendocrine tumor, clinically characterized by high blood pressure, palpitations, and headache. It is often associated with abnormalities of the ventricular repolarization phase; the dispersion of ventricular repolarization is the basis for ventricular arrhythmias (torsion de point, ventricular tachycardia or ventricular fibrillation).

**Objectives:**

Analysis of abnormal ventricular repolarization focused on the presence and amount of U wave in patients affected by pheochromocytoma and its modification after surgery.

**Materials and Methods:**

We reviewed pathology records of 722 patients admitted for adrenal nodule or suspected chromaffin-cell tumor and identified 39 patients affected by pheochromocytoma. Metanephrine, normetanephrine, and 3-methoxytyramine have been assessed by determining concentrations in 24-hour urine collection. Standard 12-lead electrocardiogram records have been reviewed with analysis of heart rate, P wave, PR interval, QRS duration, QTc, and U wave. Then we selected and compared 22 patients of 39 affected by pheochromocytoma, with both clinical and electrocardiographic data before and after surgery.

**Results:**

In our cohort of 39 patients affected by pheochromocytoma, we found U wave in ECG, before treatment, in 82.8 percent of patients, while only 37.0 percent after treatment (p<0.001) and we observed a statistically significant correlation between this wave and the urinary metanephrine. After surgery, in the selected 22 patients, we observed a clear significant reduction in systemic blood pressure, fasting glucose, metanephrine, normetanephrine, and 3-methoxytyramine. We found a significant reduction of U wave presence and leads involved in these patients after surgery (90.9% versus 9%). We observed a linear correlation between the amount of U waves in 12-lead electrocardiogram and metanephrine (r^2^=0.333, p=0.015), 3-methoxytyramine levels (r^2^=0.458, p=0.006), and tumor size (r^2^=0.429, p=0.003).

**Conclusions:**

In our retrospective analysis, patients affected by pheochromocytoma presented U wave in electrocardiogram. The presence and amount of U wave were associated with the metanephrine levels and the tumor size with significant reduction after surgical removal.

## 1. Introduction

Pheochromocytoma is a rare neuroendocrine tumor, clinically characterized by high blood pressure, palpitations, and headache. It is often associated with abnormalities of the ventricular repolarization phase; the dispersion of ventricular repolarization is the basis for ventricular arrhythmias (torsion de point, ventricular tachycardia, or ventricular fibrillation) [1].

On electrocardiogram (ECG), the U wave is the deflection following the T wave, usually of low voltage, as described for the first time by Einthoven in 1906 [2]. The U wave can be observed in different clinical conditions such as myocardial ischemia, left ventricular structural disease, acute cerebrovascular diseases, in the Andersen Tawil syndrome, multiple electrolyte disorders, hypothermia, and drug assumption [3].

Many hypotheses have been proposed to explain its genesis, based on delayed repolarization of different cardiac structure, such as the Purkinje fiber [4], papillary muscles [5], afterpotentials in a mechanoelectrical hypothesis [6], and prolonged repolarization in the cells of the mid-myocardium (“M-cells”) [7].

Previous observations have underlined pheochromocytoma-associated structural cardiomyopathy detected by echocardiographic examination and electrophysiological changing revealed by electrocardiographic analysis, in particular ventricular repolarization abnormalities, with prominent ST alterations [8].

Although the pheochromocytoma association with U waves is described in literature and descriptive studies focus on ventricular repolarization abnormalities, a comparative analysis centered on U wave analysis is missing. Our purpose is a retrospective analysis of U wave presence before and after surgical treatment of patients affected by pheochromocytoma.

## 2. Material and Methods

**Figure 1:**
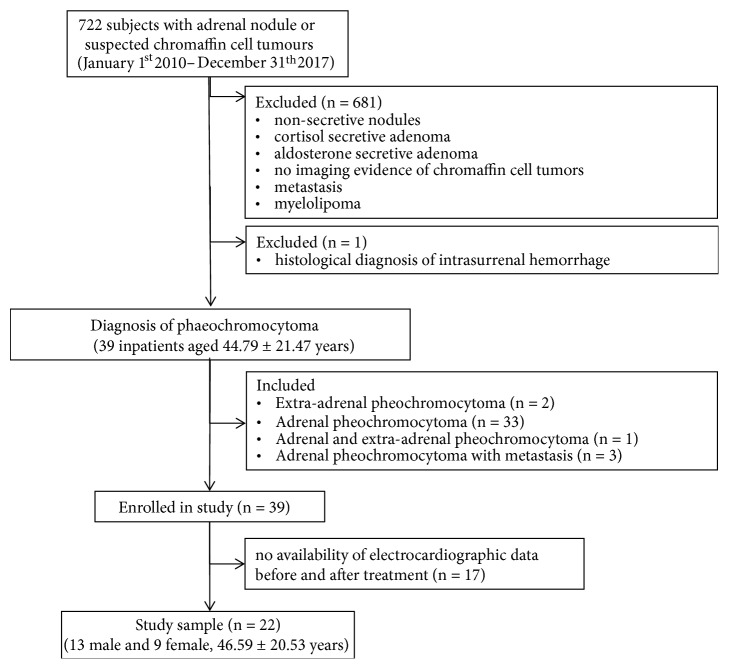
Flowchart of retrospective study design.

In this retrospective study, we reviewed clinical records of 722 patients, admitted for adrenal nodule or suspected chromaffin-cell tumor from January 1st 2010 to December 31th 2017 at “Casa Sollievo della Sofferenza” hospital (Figure 1). We excluded patients with functioning adrenal adenomas that secreted cortisol or aldosterone, nonsecretive nodules, myelolipomas, metastasis and no imaging evidence of chromaffin-cell tumor. We also excluded a patient with histological diagnosis of adrenal hemorrhage. We identified 39 patients affected by adrenal or extra-adrenal pheochromocytoma; in all of them there were high concentrations of metanephrine and/or histological diagnosis. The information on antihypertensive therapy and the size of histological tumors has been collected and the latter expressed in centimeters. Clinical patient and family history, blood pressure and biochemical data such as fasting glucose, triglycerides, cholesterol, sodium, potassium, calcium and phosphate have been gathered.

Catecholamine hypersecretion has been assessed by determining metanephrine, normetanephrine, and 3-methoxytyramine concentrations in 24-hour urine collection. The determination of plasma metanephrine and catecholamine concentrations was not available in any case, so we did not report them. Urinary excretion of metanephrines, normetanephrine, and 3-methoxytyramine has been determined in our laboratory with high performance liquid chromatography of Agilent System with normal range from 52.0 to 341.0 *μ*g/24-hours, from 88.0 to 444.0 *μ*g/24-hours, and from 103.0 to 434.0 *μ*g/24-hours, respectively.

Each standard 12-lead ECG record has been reviewed by a cardiologist who confirmed all detections reported in this study. Heart rate, P wave, PR interval, QRS duration, QTc (calculated as QT interval divided by square root of R-R interval), and U wave have been analyzed [9, 10]. We classified the patient according to the presence of U wave and the amount of leads involved on 12-lead electrocardiogram (number of U wave, range 0-12). In this retrospective study, based on revision of hospital recording, a complete echocardiographic examination fully available for each patient is missing; therefore, statistical analysis cannot be performed to explore a correlation.

Afterward, we selected 22 of 39 patients, having both clinical and electrocardiographic data before and after treatment for pheochromocytoma.

### 2.1. Statistical Analysis

Statistical analysis has been conducted using SPSS 13.0 software (Chicago, IL, USA). Continuous variables have been reported as means ± standard deviation (SD), discrete data as median and range, as well as categorical variables as percentages. Analysis of normal distribution has been performed using Kolmogorov-Smirnov test. Normetanephrine has been log-transformed (log10) before analysis. Analysis of paired values (data before and after treatment) has been performed by means of the paired* t-test* for continuous variables, the McNemar test for proportions of two dichotomous variables and Wilcoxon signed rank test for ordinal data.

Linear regression has been used to assess the correlation between two continuous data sets as between tumor dimension and urinary metanephrine.

## 3. Results

We identified 39 subjects (19 males and 20 females) with pheochromocytoma hospitalized for eight consecutive years, with a median time from symptoms to diagnosis of 6 (0-144) months (Table 1).

One patient experienced an episode of acute heart failure for myopericarditis with necessity of transfer in the Intensive Care Unit and another one has undergone an emergency surgery for retroperitoneal hemorrhage. One patient presented Takotsubo syndrome, while two other patients presented tachyarrhythmia, one of which required electrical cardioversion. Three patients had already removed the pheochromocytoma and had been hospitalized for clinical-instrumental follow-up. In three patients the detection of pheochromocytoma was an accidental detection during a magnetic resonance imaging performed to control other neoplasms. In eight cases the pheochromocytoma has been diagnosed during a family screening for cancer, such as neurofibromatosis type 1 and multiple endocrine neoplasia type 2. We found the metastasis of malignant pheochromocytoma in three subjects who had arrived at the emergency department for hypertensive crisis and abdominal pain.

We observed seven subjects with pheochromocytoma and hypertensive crisis, sweating, palpitations, and anxious state; one of them, inoperable, was treated with Iodine-131 metaiodobenzylguanidine therapy. Four patients with previous excision of pheochromocytoma have been admitted for follow-up. Symptoms have not been reported in the medical record for the other patients.

In all this set of patients, we found U wave at ECG analysis before treatment in 82.8 percent of patients while only in 37.0 percent after treatment (p<0.001) and we observed a statistically significant correlation between U wave presence and the urinary metanephrine (3614.60 ± 4906.55 *μ*g/24-hours in presence of U wave versus 855.40 ± 680.87 *μ*g/24-hours without U wave, p=0.031).

Pharmacological therapy is reported in Table 2; according to data showed, we observed a clear reduction of alpha blocker agents after cancer treatment, in particular almost total removal of phenoxybenzamine and halved prescription of alfuzosin.

**Figure 2:**
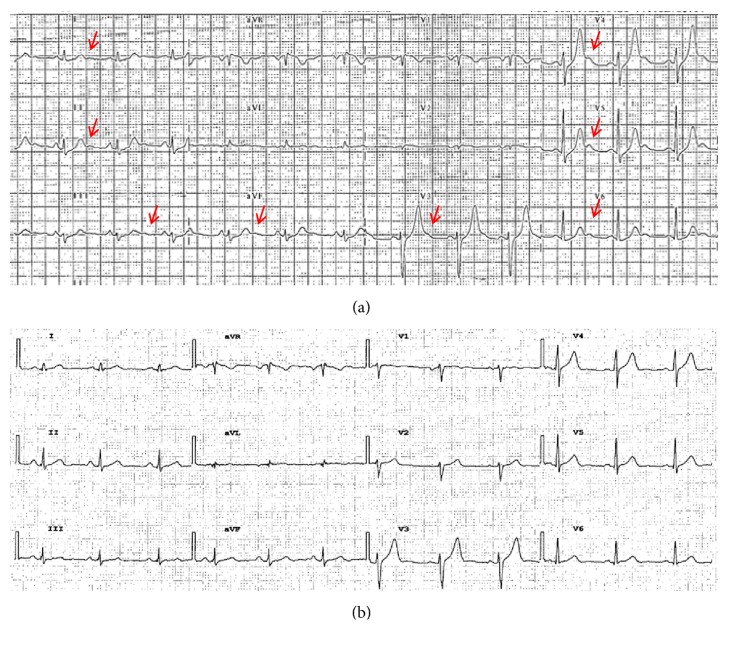
*Example of 12 lead ECG in the same patient before and after treatment*. (a) ECG at hospitalization. The arrows in red highlight the presence of U wave in D1, D2, D3, aVF, V3-V6 leads. (b) ECG four months after surgery. No evidence of U wave.

**Figure 3:**
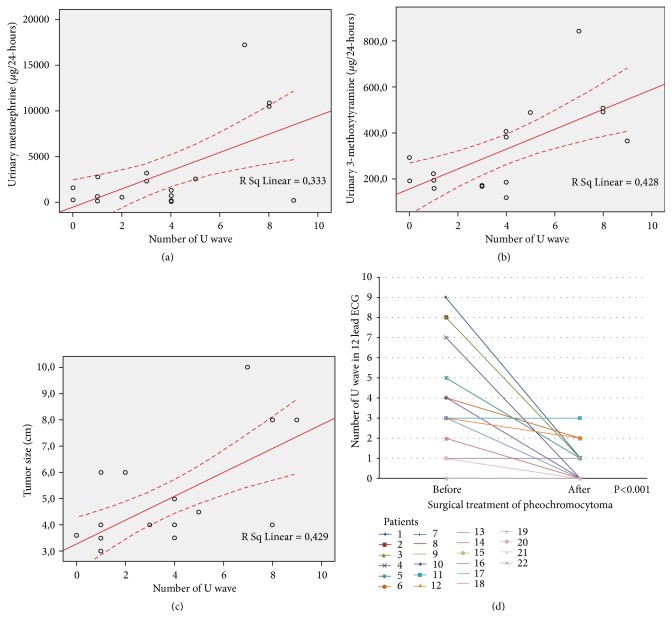
*Correlation between U wave amount and levels of urinary metanephrine, 3-methoxytyramine and tumor size and reduction of U wave after surgery treatment*. (a) Concentrations of urinary metanephrine plotted against number of U wave. (b) Concentrations of urinary 3-methoxytyramine plotted against U wave amount. (c) Tumor size plotted against U wave amount. (d) Reduction of U wave amount after surgery in each patients. Linear regression was performed in (a), (b),and (c) plots, and the r^2^ value was shown for each analysis. Wilcoxon signed rank test was performed in (d) plot, and the p value was shown.

Standard 12-lead ECG before and after surgery was available in only 22 patients. All these patients were in sinus rhythm, without any bundle branch block. After surgery, according to expectations, we observed a clear significant reduction in systemic blood pressure, fasting glucose, metanephrine, normetanephrine and 3-methoxytyramine and decreased antihypertensive drugs assumption (Table 3). Heart rate, PR interval and QTc decreased statistically significantly after surgery. There was no difference in terms of triglycerides, cholesterol, electrolytes, duration of P wave, and QRS. We found clear presence of U wave in 20 patients (90.9%) before surgery, compared to 9 patients (42.9%) after treatment (p=0.002) (Figure 2). We observed a significative reduction in U wave amount after surgery (3 [range 0-9] versus 0 [range 0-3], respectively before and after treatment, p<0.001) (Figure 3). We found a linear correlation between U wave amount in 12-lead ECG and metanephrine (r^2^=0.333; p=0.015), 3-methoxytyramine levels (r^2^=0.458, p=0.006) and tumor size (r^2^=0.429, p=0.003) (Table 4). Moreover, we observed a linear correlation among tumor dimension, metanephrine levels and U wave amount, although one outlier was likely due to dimensional overestimation as a result of fatty tissue infiltration. A linear correlation was also found between tumor size and normetanephrine (r^2^=0.378, p=0.019) and between tumor size and 3-methoxytyramine (r^2^=0.627, p=0.001).

## 4. Discussion

The present study retrospectively analyzed pheochromocytoma-associated clinical detection in a cohort of 39 patients, equally distributed among gender, derived from 722 reviewed clinical records, hospitalized for adrenal nodule or suspected of chromaffin-cell tumors.

Although the wide spectrum of clinical presentation spread from asymptomatic patient to cardiogenic shock, median time for diagnosis since symptoms onset is 6 months, going from a clear initial diagnostic detection to a period of 144 months of frequent hospitalizations; even if not statistically significant, we found an inverse relationship between metanephrine levels and much more time for identification (data not showed) maybe related to mild symptoms.

In more than 25% of cases, patient had a family history of cancer. In particular, over 20% of patients had characteristic familial endocrine neoplastic history, such as neurofibromatosis type 1 (5.1%), multiple endocrine neoplasia type 2 (10.3%), pheochromocytoma (2.6%), and Von Hippel-Lindau syndrome (2.6%); according to recent guidelines, in presence of these family history and hypertension, particularly in young adults, clinicians have to rule out pheochromocytoma [11].

In our analysis, pheochromocytoma had a malignant course, with capsule infiltration (12.8%), recurrence (7.7%), and metastasis (7.7%), underlying the clinical relevance of an earlier diagnosis and systematic evaluation.

Cardiogenic shock and Takotsubo syndrome depicted the acute dramatic presentation in two patients, both rapidly stabilized and referred to surgical treatment.

Arrhythmias have been found in only two patients, characterized by atrial fibrillation and junctional tachycardia; this low prevalence was probably due to systemic administration of both preoperative alpha- and beta-blocker therapy.

Despite previous descriptions, in our patients we did not find prominent ST alteration; therefore, we analyzed all ECG intervals and U wave presence; one explanation relies on the months from the initial symptoms to diagnosis, that in the case of the population examined by Giavarini et al. [12] was about seven years, longer than our detection, as hypertension was reported at mean age of 40 years and diagnosis was made at mean age of 47 years, allowing major cardiac abnormality in the meantime.

Physiopathological model of U wave genesis in patient affected by pheochromocytoma could bring together all historical perspectives; increase in systemic blood pressure and heart rate will overstretch cardiac cells, probably increasing microcirculatory dysfunction and ischemia, with subsequent papillary muscle delayed repolarization and after potential generation, as suggested by mechanoelectrical hypothesis; as shown in our detection, U wave is strictly associated with blood pressure value, the last one playing a questionable role as a confounding factor or a mechanical determinant. At the same time catecholamine-mediated effects prolong action of potential duration on both Purkinje fibers and cardiac M-cells, delaying the repolarization period [13, 14].

According to Sir Austin Bradford Hill [15], our investigation points out the plausibility of cause-effect relationship between metanephrine levels and U wave presence; in particular, we have demonstrated the strength of association between U wave presence and disease, biochemical gradient between metanephrine levels, tumor size and U wave amount, reversal temporality between U wave reduction and cancer removal. All the above factors have been supported by biological plausibility and clinical coherence, already underlined by the previous four mentioned theories.

Pharmacological treatment could potentially affect cardiac repolarization; for these reason we have collected all pharmacological therapy before and after cancer removal. We have noticed a clear reduction in alpha blocker prescription, without significant variation among other drugs, while nearly 54% of patients have discontinued all antihypertensive therapy after cancer treatment. Although in scientific literature it has been described that alfuzosin may prolong QT interval at high dosage via sodium current increase, there are no descriptions on U wave induction by this drug [16]. On the other side, also if phenoxybenzamine may potentially delay cardiac repolarization, yet a case report described QT prolongation and ischemia-related giant T wave reversed by sodium nitroprusside, unaffected by phenoxybenzamine administration in a patient with pheochromocytoma [17]. Finally, alpha blocker administration could be a confounder factor for electrocardiographic analysis in pheochromocytoma, as almost all patients are treated by this drug since diagnosis with subsequent removal after surgery, preventing a possible study with placebo in the future for ethical issue.

This retrospective study, based on revision of hospital recording, is missing a complete echocardiographic examination fully available for each patient; this represents a limit as cardiac repolarization may be affected by presence of structural heart disease. Indeed, as we are comparing the same patient, before and after surgery, after a short period, the possible presence of permanent structural disease should not affect temporary difference in ventricular repolarization, pointing out the plausible effect of cancer removal and catecholamine reduction on electrocardiographic findings.

In conclusion, a careful analysis of the electrocardiogram, particularly the phase of ventricular repolarization characterized by U wave, is mandatory in patients presenting a clinical scenario characterized by diaphoresis, headache, palpitation, and hypertensive crisis; this detection should lead the clinicians to suspect the diagnosis of pheochromocytoma, lightening the initial doubtful clinical presentation, especially for endocrine neoplastic family history.

## 5. Conclusions

Our observations suggest the U wave presence at ECG analysis of patients presenting with hypertensive crisis, palpitation, headache, and diaphoresis should lead the clinician to suspect the diagnosis of pheochromocytoma; according to modern clinical approach, there is not a* single winner player* finding in disease diagnosis; yet the job of a physician would always proceed collecting several clues to reach successful patient management; as highlighted by Sir Arthur Conan Doyle, “*it has long been an axiom that the little things are infinitely the most important*”.

## Figures and Tables

**Table 1:** Clinical data of patients with phaeochromocytoma.

Clinical data	Pheochromocytoma
	(n = 39)
Age, mean (SD), y	44.79 ± 21.47

Sex, No. (%)	
Female	20 (51)
Male	19 (49)

Months of symptoms, median (range), m	6 (0-144)

Age at first treatment, mean (SD), y	43.03 ± 21.55

Tumor size, mean (SD), cm	5.04 ± 2.52

Tumor type or location, No. (%)	
Adrenal	36 (92.3)
Right side	18 (50.0)
Left side	18 (50.0)
Extra-adrenal	3 (7.7)
Infiltration of the capsule	5 (12.8)
Metastasis	3 (7.7)
Recurrence of adrenal and extra-adrenal	3 (7.7)

Comorbidities, No. (%)	
None	10 (25.6)
Hypertension	29 (74.4)
Dyslipidemia	6 (15.4)
Diabetes	5 (12.8)
Cardiogenic shock	2 (5.1)
Paroxysmal supraventricular tachycardia	2 (5.1)
Hypothyroidism	6 (15.4)
Other malignant tumors	4 (10.3)
Other benign tumors	4 (10.3)

Family history of cancer, No. (%)	
Neurofibromatosis type 1	2 (5.1)
Multiple endocrine neoplasia type 2	4 (10.3)
Phaeochromocytoma	1 (2.6)
Von Hippel-Lindau syndrome	1 (2.6)
Other types of neoplasia	2 (5.1)

History of structural heart disease, No. (%)	
Absence	24(61.5)
Hypertensive heart disease	10(25.6)
Ischemic heart disease	4(10.3)
Valvular heart disease	1(2.6)

**Table 2:** Pharmacological treatment before and after cancer removal in 39 patients.

Treatment before removal, No. (%)	
Alpha blocker	34(87.2)
*Alfuzosin*	23(59)
*Phenoxybenzamine*	11(28.2)
Beta blocker	11(28.2)
Calcium channel blocker	4(10.3)
Angiotensin II Receptor Blockers/ACE inhibitors	3(7.7)
Diuretics	2(5.1)

Treatment after removal, No. (%)	
Alpha blocker	11(28.2)
*Alfuzosin*	10(25.6)
*Phenoxybenzamine*	1(2.5)
Beta blocker	8(20.5)
Calcium channel blocker	1(2.5)
Angiotensin II Receptor Blockers/ACE inhibitors	4(10.3)
Diuretics	3(7.7)
None	21(53.8)

**Table 3:** Biochemical data and electrocardiographic parameters of 22 patients before and after pheochromocytoma treatment.

	Before treatment	After treatment	*P* value
Systolic blood pressure (mmHg)	149.28 ± 23.07	117.33 ± 13.16	<0.001
Diastolic blood pressure (mmHg)	87.72 ± 14.21	74.22 ± 13.34	0.006
Fasting glucose (mg/dl)	109.41 ± 36.79	83.95 ± 11.75	0.002
Triglycerides (mg/dl)	106 ± 42.05	95.06 ± 37.39	0.291
Cholesterol (mg/dl)	195.93 ± 53.83	194.07 ± 52.72	0.878
Sodium (mmol/l)	142,09 ± 2.62	141.68 ± 2.59	0.638
Potassium (mmol/l)	4.36 ± 0.53	4.29 ± 0.44	0.512
Calcium(mg/dl)	9.21 ± 1.09	9.15 ± 0.88	0.783
Phosphate (mg/dl)	3.86 ± 1.03	3.48 ± 0.82	0.188
Metanephrine (*μ*g/24-hours)	3808.55 ± 5491.49	136.76 ± 83.28	0.032
Normetanephrine (*μ*g/24-hours)	5979.41 ± 8123.95	266.53 ± 108.83	<0.001
3-methoxytyramine (*μ*g/24-hours)	373.19 ± 206.25	196.10 ± 131.74	0.047
P wave (msec)	101.60 ± 18.43	96.80 ± 18.08	0.350
PR (msec)	145.06 ± 25.68	157.56 ± 33.10	0.007
QRS (msec)	84.20 ± 17.26	85.50 ± 12.93	0.705
QTc (msec)	425.20 ± 30.72	405.40 ± 29.76	0.019
Heart rate (bpm)	78.81 ± 15.56	70.86 ± 11.66	0.067
Presence of U wave, No. (%)	20 (90.9)	9 (42.9)	0.002^a^
Number of U wave in 12 lead ECG, median (range)	3 (0-9)	0 (0-3)	<0.001^b^
Antihypertensive therapy, No. (%)	1.67 ± 1.46	0.59 ± 0.71	0.007

*P *values are for paired-samples *t* test.

^a^ *P* values is for McNemar test.

^b^ *P* values are for Wilcoxon signed rank test.

**Table 4:** Linear correlations of U wave amount in 12-lead ECG and tumor size.

	Beta	r^2^	*P* value
*Number of U wave*			
Systolic blood pressure (mmHg)	0.494	0.244	0.031
Diastolic blood pressure (mmHg)	0.624	0.390	0.004
Fasting glucose (mg/dl)	0.122	0.015	0.599
Metanephrine *μ*g/24-hours	0.557	0.333	0.015
Normetanephrine *μ*g/24-hours	0.404	0.163	0.108
3-methoxytyramine *μ*g/24-hours	0.654	0.428	0.006
P wave (msec)	-0.127	0.016	0.628
PR (msec)	-0.065	0.004	0.791
QRS (msec)	0.280	0.078	0.232
QTc (msec)	-0.001	1.605^−6^	0.996
Heart rate (bpm)	0.037	0.001	0.872

*Tumor size*			
Systolic blood pressure (mmHg)	0.081	0.007	0.765
Diastolic blood pressure (mmHg)	0.112	0.013	0.679
Fasting glucose (mg/dl)	0.077	0.005	0.760
Metanephrine (*μ*g/24-hours)	0.615	0.378	0.019
Normetanephrine (*μ*g/24-hours)	0.893	0.798	<0.001
3-methoxytyramine (*μ*g/24-hours)	0.792	0.627	0.001
P wave (msec)	0.065	0.004	0.818
PR (msec)	0.093	0.009	0.722
QRS (msec)	-0.022	4.759^−4^	0.932
QTc (msec)	-0.061	0.004	0.809
Heart rate (bpm)	0.238	0.057	0.341
Presence of U wave	0.655	0.429	0.003

## Data Availability

Our data have been obtained by careful analysis of Casa Sollievo della Sofferenza Hospital clinical recordings.

## Abbreviations

ECG: Electrocardiogram
SD: Standard Deviation.
